# TCR signals fuel T_reg_ cells

**DOI:** 10.18632/oncotarget.4881

**Published:** 2015-07-12

**Authors:** Christoph Drees, J. Christoph Vahl, Marc Schmidt-Supprian

**Affiliations:** Hematology and Oncology, Klinikum rechts der Isar, Technische Universität München, Munich, Germany

**Keywords:** Immunology and Microbiology Section, Immune response, Immunity

Regulatory T (T_reg_) cells safeguard against autoimmunity and overshooting inflammation. During their development in the thymus, T_reg_ cells are selected by stronger autoantigenic T cell receptor (TCR) signals than conventional T cells. These TCR signals are critically important for the initiation of two key lineage defining events, namely induction of the transcription factor Foxp3 and hypomethylation of a specific gene set. Recent studies shed light on the role of continuous (autoreactive) TCR signals for identity, homeostasis and functions of mature T_reg_ cells.

Induced TCR ablation on mature T_reg_ cells only minimally reduces Foxp3 expression [1, 2] and does not affect hypomethylation patterns [1]. Stable Foxp3 expression was also observed in T_reg_ cells lacking the TCR signal transduction proteins Lck [3] or SLP-76 [4] and upon ablation of the co-stimulatory receptor CD28 [5]. Fittingly, mature T_reg_ cells, expressing a TCR but deprived of peripheral autoantigenic stimulation due to lack of MHC II on hematopoietic cells, still express Foxp3 [6]. Therefore, once a core T_reg_ cell identity has been established in the thymus, it is maintained independently of peripheral TCR signals.

In contrast, the T_reg_ cell surface phenotype and their signature gene expression were strongly affected by the various means of inhibiting TCR signals [1–3, 6]. However, the peripheral T_reg_ cell pool is heterogeneous in that it consists of naïve and various subsets of effector-like T_reg_ cells. Induced TCR ablation showed that, at least under homeostatic conditions in healthy mice, effector-like T_reg_ cells strictly depend on TCR signals for their generation and/or maintenance. Attempts to distinguish between naïve and effector-like T_reg_ cells based on CD62L/CD44 [2] or CD25 [1] expression revealed that both require TCR signals to maintain their characteristic surface phenotype and gene expression, independently of their homeostasis. Interestingly, apart from reduced levels of TCR-activated transcription factors such as Egr2 and c-Rel and their respective target genes, loss of TCR signals strongly affects IRF4-controlled genes. The concept that DNA hypomethylation ensures the expression of key T_reg_ cell genes is supported by the reduced, but still robust expression of CTLA-4, GITR and Eos of TCR-deficient T_reg_ cells [1].

Both induced TCR and co-stimulatory CD28 ablation cause a decline in T_reg_ cells in the absence of thymic T cell production, which goes hand in hand with completely abrogated [1] or reduced [5] homeostatic proliferation, respectively. This is not due to reduced responsiveness to homeostatic cytokines, such as IL-2. Interestingly, T_reg_ cells deprived of MHC II contact proliferate well in response to anti-CD3 stimulation [6] and TCR-deficient T_reg_ cells divide when stimulated with TCR-bypassing PMA/Ionomycin [2], indicating that these cells do not become anergic to proliferation-inducing signals. The protein levels of Bcl-2 and Bim are two-fold upregulated in T_reg_ cells upon TCR loss, to the same levels as in naïve CD4 T cells [1]. However, none of the studies reported a significant difference in survival between TCR signaling impaired and normal T_reg_ cells *in vivo*, and BrdU pulse-chase experiments suggest impaired turn-over/proliferation, but normal survival of Lck-deficient T_reg_ cells [3]. Collectively, these experiments show that TCR signal-induced proliferation forms an essential homeostatic requirement for all T_reg_ cell subsets.

T_reg_ cells suppress immune responses through the release of inhibitory cytokines, competition for IL-2, access to and functional modulation of antigen presenting cells and direct cytotoxic killing. TCR-deficient T_reg_ cells show reduced expression of inhibitory and cytotoxic molecules including IL-10 and Granzyme B. In addition, the expression of proteins through which T_reg_ cells regulate the functions of antigen presenting cells, such as CTLA-4, NT5E/CD73, LFA-1 and NRP1, is significantly reduced [1, 2, 6]. TCR signaling impaired T_reg_ cells fail to efficiently suppress T cell activation and proliferation *in vitro* [3–6]. Conversely, augmenting TCR signaling by ablating the DAG-metabolizing kinase DGKζ enhances the *in vitro* suppression capacity [4]. Importantly, T_reg_ cells require constant TCR signals to sustain their suppressive abilities *in vivo*: MHC II contact-deprived T_reg_ cells fail to control naïve T cell expansion upon co-transfer [6], TCR-deficient T_reg_ cells cannot control effector T cell differentiation/proliferation and cytokine production *in situ* [2] and induced TCR ablation limits T_reg_ cell-mediated control of colitis and EAE [1]. Therefore, TCR signals continuously arm T_reg_ cells for suppression, in addition to their role in their homeostasis.

Expression height of Ly-6C, which is strongly elevated in Lck-deficient [3] and TCR-deficient [1] T_reg_ cells, appears to differentiate T_reg_ cells receiving strong TCR signals (Ly-6C−) from those that do not (Ly-6C+) [7]. Purified Ly-6C− and Ly-6C+ T_reg_ cells differed dramatically in phenotype, gene expression and their ability to suppress *in vitro* and *in vivo*. Accordingly, effector T_reg_ cells are exclusively Ly-6C−. However, on average half of the TCR-deficient T_reg_ cells lacked Ly-6C (Vahl et al., unpublished), showing that not all Ly-6C− T_reg_ cells result from continuous TCR triggering. Modern multiparameter flow cytometry should help to further unravel the dynamic complexities of T_reg_ cell subsets.

In summary, we conclude that while TCR signals are dispensable for maintaining the core T_reg_ cell identity, they are continuously fueling their homeostasis, effector differentiation and suppressive functions.
